# Salvageable Intra-stent Coil Embolization Treatment for Spinal Dural Arteriovenous Fistula in a Child

**DOI:** 10.7759/cureus.42425

**Published:** 2023-07-25

**Authors:** Abdullah S Almawi, Abdulaziz S Alhammad, Zaid A Alshammari, Shorog Althubait, Sultan Alqahtani

**Affiliations:** 1 Radiology, Security Forces Hospital, Riyadh, SAU; 2 Radiology, King Faisal Specialist Hospital and Research Centre, Riyadh, SAU; 3 Radiology, King Fahad Medical City, Riyadh, SAU; 4 Interventional Neuroradiology, King Fahad Medical City, Riyadh, SAU

**Keywords:** interventional neuroradiology, spinal, pediatric, endovascular embolization, arteriovenous fistulas

## Abstract

Spinal dural arteriovenous fistulae (SDAVF) are rare diseases that exhibit abnormal connections between arteries and veins. They are even rarer in the pediatric population and pose diagnostic and treatment challenges for physicians. Its presentation varies depending on the site and size of the SDAVF. Multiple management options are available, which are usually tailored depending on the patient's condition. Here, we present a rare case of SDAF in a four-year-old girl who initially presented with bilateral lower limb weakness. The patient was then treated successfully using primary major fistula point stenting and intra-stent coiling, with complete closure achieved. Full recovery was achieved over the course of follow-ups. The deep analysis of SDAVF, its classification, and the utilization of the best available endovascular tools by a dedicated neurovascular team offer the best outcome in dealing with complex spinal neurovascular pathologies.

## Introduction

Spinal dural arteriovenous fistulae (SDAF) are extremely rare in the pediatric population, and only a few case reports have been reported [1-6]. Moreover, the presentation of such patients poses a diagnostic challenge to treating physicians. The symptoms include neurological deficits such as impaired sensation, paralysis, disturbed micturition, and defecation [4,7,8]. Moreover, patients may present with back pain due to subarachnoid hemorrhage [7,8]. 

It is always challenging to treat SDAFs, especially the complex multichannel connections between arteries and veins without an intervening capillary bed. The high arterial blood flow pooling into the veins may cause hemorrhage, or vessels may grow and cause a mass effect compressing the spinal cord, spinal canal, and bone remodeling and widening due to long-standing pulsatile effects [9,10].

There are four reported types of spinal arteriovenous malformations (AVMs) i.e., type I spinal dural AVMs, which are slow-flow lesions that mostly occur in adulthood; type II spinal glomus AVMs, which are congenital and considered high-flow lesions; type III spinal juvenile AVMs, which form prenatally and are high-flow lesions; and type IV intradural perimedullary AVMs, which are pia-based lesions [8,11,12]. We report a rare case of type IV SDAVF in a child who was treated with endovascular embolization.

## Case presentation

**Figure 1:**
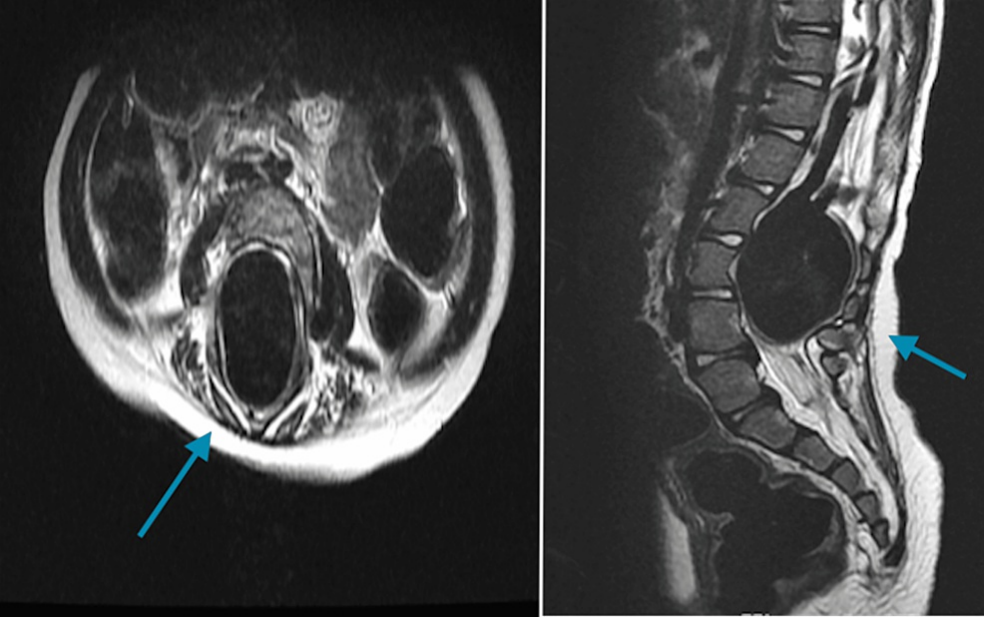
Axial (left) and sagittal (right) T2 weighted images of the lumbar spine

Our patient was a four-year-old girl known to have obstructive hydrocephalus due to congenital aqueductal stenosis. She had been on a ventriculoperitoneal (VP) shunt since she was six months old. The patient complained of progressive bilateral lower limb weakness for six months with intact defecation and micturition functions. She was referred to our institute for further investigation and management. Upon examination, the patient had severe symmetrical weakness of the lower extremities. However, her neurological functions in her upper limbs were intact. An urgent MRI of the whole spine revealed multiple shunts with marked intradural aneurysmal venous varix at levels L2, L3, and L4 (Figure 1). The aneurysmal varix was causing significant widening of the spinal canal, severe compression of the neural elements, scalloping at the posterior aspect of the vertebral bodies, and erosions of the posterior neural arch. 

**Figure 2:**
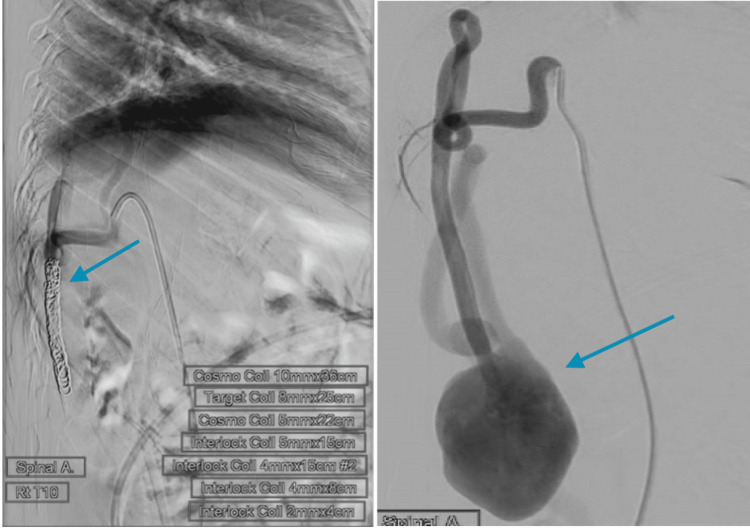
Digital subtraction angiography pre (right) and post (left) stent placement, and embolization with coils at the lumbar spine area

The neurovascular team decided to go for a complete spinal angiogram to identify the location, type, and best possible treatment options. The spinal angiogram showed a large multi-channel SDAVF with large shunting (Figure 2). Initial attempts to occlude the major fistula point with coils alone were not possible due to the high flow velocity of arteriovenous shunting and the smooth lumen of the vessel. So, we elected to deploy a stent along the distal arterial point to create a rough surface for the coils to engage. Complete occlusion of the major fistula point was achieved successfully with intra-stent coiling, and the rest of the smaller minor shunts were occluded using coils and onyx liquid embolic agent (Figure 2).

**Figure 3:**
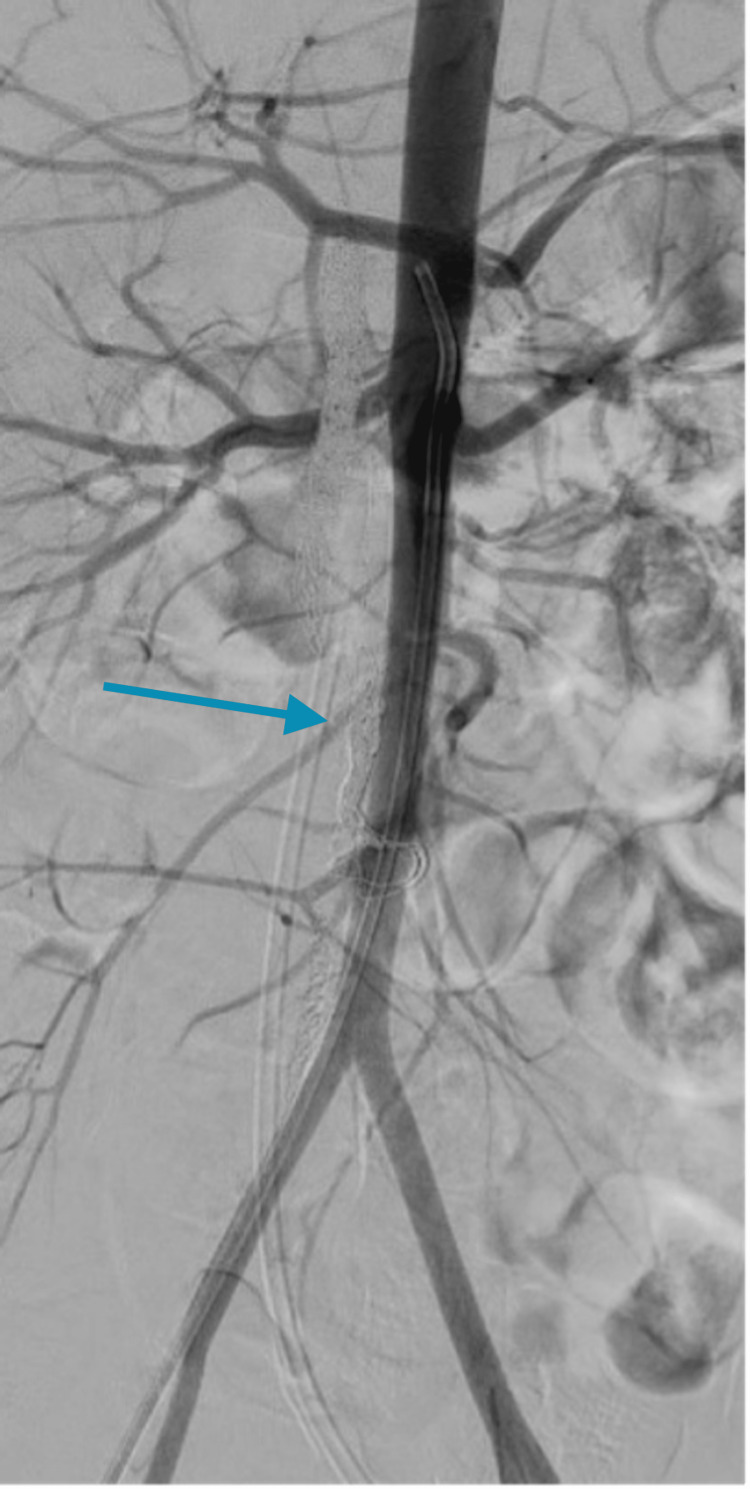
Digital subtraction angiography of the aorta

The patient tolerated the procedure well with no complications. She was discharged two days later in good condition. The patient was on continuous follow-up at the pediatric neurology clinic and was placed on a rehabilitation program post-procedure. The patient demonstrated significant progressive improvement in 24 months, and she made an almost full recovery. A follow-up aortogram after two years showed complete SDAVF closure with no residual (Figure 3).

## Discussion

Early detection of spinal SDAVF is crucial to alleviating its symptoms and ensuring complete recovery. It’s also important to prevent rupture and minimize hemodynamic jeopardy. However, the pediatric population needs delicate planning to weigh the risks and benefits of endovascular interventions, including the use of angiography, radiation-related hazards, and general anesthesia [13,14]. 

The decision to undertake endovascular or open vascular intervention must be preceded by a comprehensive understanding of the lesion with a methodological approach and utilization of imaging (MRI and angiograms) to determine the complexity of the lesion, its size, and feeding arteries. For instance, the risk of rupture increases with larger lesions. Hence, open vascular interventions won’t be optimal in such situations [15]. Another option is the combination of endovascular and open vascular interventions, which would help in ensuring complete resection and occlusion [1,9]. Sometimes it is also required to undergo both endovascular and open vascular interventions at multiple stages [9]. 

In many cases, endovascular intervention is considered a suitable option due to its minimally invasive approach, accuracy of anatomical localization, and selective catheterization of vessels [16]. Many cases have reported complications and postoperative deterioration in patients who underwent open vascular interventions [12]. However, this may be related to the fact that patients who undergo open vascular interventions are often more complex and have already attempted endovascular interventions. Complications of endovascular interventions include hemorrhage, dissection of vessels, spasm, or, in more severe cases, the occlusion of vessels and ischemia [8,14]. This sheds light on the importance of preoperative planning and the presence of a multidisciplinary team to minimize the risk of complications. For example, the involvement of anesthesia to prepare for hemodynamic changes perioperatively.

The prognosis of such interventions may be affected by many factors, such as a patient's comorbidities, hemodynamic stability, and postoperative complications. Patients with high-flow lesions are prone to permanent neurological deficits [15]. And patients with incomplete treatment are susceptible to worsening of clinical symptoms and recurrence of hemorrhage [5,6]. Thus, ensuring complete occlusion is important to avoid such results. Especially in pediatric patients, as they have small feeding vessels, which makes complete occlusion a challenge [17]. So, using a suitable approach with competent expertise assures optimal results. A case series reported that most patients who underwent endovascular interventions had improved symptoms on follow-ups [16]. In unfortunate cases requiring another stage of intervention, such as in our case, monitoring with clinical examination and imaging is essential to making an informed decision on the next step of management. 

This case report highlights the importance of preoperative planning and utilizing the proper technique and expertise, as well as clinical and imaging follow-ups, to ensure optimal results for such patients. More research and prospective studies are needed to classify the patients and help in choosing the most suitable management plan, i.e., an endovascular intervention, an open vascular intervention, or a combination of both. 

## Conclusions

Spinal dural arteriovenous fistulae, being a rare entity in children, crucially require early detection, proper evaluation, and characterization of such lesions prior to the initiation of management options. It is essential to have the availability of a highly specialized and well-integrated neurovascular team as well as other teams involved, such as anesthesia. In the presence of all treatment options, this will hopefully enhance the treatment success rate for such complex neurovascular cases. More prospective studies are needed to stratify patients and help choose the most suitable management option.

## References

[1] Kalani MY, Ahmed AS, Martirosyan NL (2012). Surgical and endovascular treatment of pediatric spinal arteriovenous malformations. World Neurosurg.

[2] Rodesch G, Hurth M, Alvarez H, Ducot B, Tadie M, Lasjaunias P (2004). Angio-architecture of spinal cord arteriovenous shunts at presentation. Clinical correlations in adults and children. The Bicêtre experience on 155 consecutive patients seen between 1981-1999. Acta Neurochir (Wien).

[3] Cullen S, Alvarez H, Rodesch G, Lasjaunias P (2006). Spinal arteriovenous shunts presenting before 2 years of age: analysis of 13 cases. Childs Nerv Syst.

[4] Du J, Ling F, Chen M, Zhang H (2009). Clinical characteristic of spinal vascular malformation in pediatric patients. Childs Nerv Syst.

[5] Antonietti L, Sheth SA, Halbach VV (2010). Long-term outcome in the repair of spinal cord perimedullary arteriovenous fistulas. AJNR Am J Neuroradiol.

[6] Lee YJ, Terbrugge KG, Saliou G, Krings T (2014). Clinical features and outcomes of spinal cord arteriovenous malformations: comparison between nidus and fistulous types. Stroke.

[7] Rodesch G, Pongpech S, Alvarez H, Zerah M, Hurth M, Sebire G, Lasjaunias P (1995). Spinal cord arteriovenous malformations in a pediatric population children below 15 years of age the place of endovascular management. Interv Neuroradiol.

[8] Bertoli MJ, Parikh K, Klyde D, Mazzola CA, Pandya Shah S (2021). Spinal arteriovenous malformation in a pediatric patient with a history of congenital syphilis: a case report. BMC Pediatr.

[9] Bagherpour AN, Rodriguez GJ, Moorthy C, Trier TT, Maud A (2016). Combined surgical and endovascular treatment of complex high-flow conus medullaris arteriovenous fistula associated with Parkes Weber syndrome: case report. J Neurosurg Spine.

[10] Wilson DA, Abla AA, Uschold TD, McDougall CG, Albuquerque FC, Spetzler RF (2012). Multimodality treatment of conus medullaris arteriovenous malformations: 2 decades of experience with combined endovascular and microsurgical treatments. Neurosurgery.

[11] Kona MP, Buch K, Singh J, Rohatgi S (2021). Spinal vascular shunts: a patterned approach. AJNR Am J Neuroradiol.

[12] Zhang HJ, Silva N, Solli E, Ayala AC, Tomycz L, Christie C, Mazzola CA (2020). Treatment options and long-term outcomes in pediatric spinal cord vascular malformations: a case report and review of the literature. Childs Nerv Syst.

[13] Saul D, Mong A, Biko DM (2016). Pediatric considerations in computed tomographic angiography. Radiol Clin North Am.

[14] Hoffman CE, Santillan A, Rotman L, Gobin YP, Souweidane MM (2014). Complications of cerebral angiography in children younger than 3 years of age. J Neurosurg Pediatr.

[15] Cho WS, Wang KC, Phi JH (2016). Pediatric spinal arteriovenous malformations and fistulas: a single institute's experience. Childs Nerv Syst.

[16] Consoli A, Smajda S, Trenkler J, Söderman M, Rodesch G (2019). Intradural spinal cord arteriovenous shunts in the pediatric population: natural history, endovascular management, and follow-up. Childs Nerv Syst.

[17] Cho WS, Kim KJ, Kwon OK, Kim CH, Kim J, Han MH, Chung CK (2013). Clinical features and treatment outcomes of the spinal arteriovenous fistulas and malformation: clinical article. J Neurosurg Spine.

